# Adverse reactions of BNT162b2 vaccine booster against COVID‐19 in Japan

**DOI:** 10.1002/jgf2.545

**Published:** 2022-04-06

**Authors:** Toshinori Nishizawa, Torahiko Jinta, Ryosuke Koyamada, Yuki Uehara, Fumika Taki, Hiroko Arioka

**Affiliations:** ^1^ Department of General Internal Medicine St. Luke's International Hospital Tokyo Japan; ^2^ Department of Pulmonary Medicine Thoracic Center, St. Luke's International Hospital Tokyo Japan; ^3^ Department of Hematology St. Luke's International Hospital Tokyo Japan; ^4^ Department of Infectious Diseases St. Luke's International Hospital Tokyo Japan; ^5^ Department of Clinical Laboratory Center St. Luke's International Hospital Tokyo Japan; ^6^ Department of Nephrology St. Luke's International Hospital Tokyo Japan

## Abstract

Female participants had a higher incidence of headache, nausea, myalgia, arthralgia, redness, pruritus, and inoculation site redness and pruritus (p 0.05). Low grade fever, headache, malaise, myalgia, and inoculation site induration and heat were associated with age group (p 0.05).

To the Editor,

On December 3, 2021, the administration of the third dose of the BNT162b2 messenger RNA vaccine (Pfizer‐BioNTech) was started in Japan for individuals who had received a second dose of the vaccine at least 8 months earlier. Clinical research in the United States, Israel, and other countries has already proven its efficacy and short‐term safety; however, the adverse reactions in Japanese people after the third dose of the vaccine were unknown.^1^, ^2^

From December 3, 2021, to December 18, 2021, 1880 healthcare workers received the third dose in our hospital. A self‐administered email questionnaire was then sent to these participants. Of the total participants, 649 participants (508 [78%] female) agreed to participate in this study to survey the frequency of adverse reactions to vaccines. This research project was prepared before the third immunization campaign. The protocol of this study was approved by the Institutional Ethics Committee with registration No. 21‐R144. All the participants have already been vaccinated with the first and second dose of the BNT162b2 messenger RNA vaccine (Pfizer‐BioNTech). The age distribution was as follows: 19.8% of participants were in their 20s, 28.2% in 30s, 28.2% in 40s, 18.3% in 50s, and 5.6% in 60s. After the third dose, 646 participants (99.5%) experienced some adverse reaction, regardless of gender and age. Adverse reactions included high‐grade fever (≧38°C, 21.6%), low‐grade fever (37.0°C–37.9°C, 54.9%), headache (59.3%), malaise (78.2%), nasal discharge (6.2%), nausea (13.7%), vomiting (1.9%), diarrhea (7.1%), chills (49.5%), myalgia (61.3%), and arthralgia (42.8%) and pain (92.3%), redness (16.6%), swelling (25.7%), induration (10.0%), pruritus (15.0%), and heat (32.2%) at the inoculation site. Female participants had a higher incidence of headache, nausea, myalgia, arthralgia, redness, pruritus, and inoculation site redness and pruritus (*p* < 0.05, Table 1). High‐grade fever, headache, malaise, and myalgia were reported significantly more frequently in the younger age groups (*p* < 0.05, Table 1). The incidence of these adverse reactions decreased with age. Antipyretics were taken by 67.6%. Female participants significantly tended to use antipyretics (*p* < 0.05; odds ratio, 1.57; 95% confidence interval, 1.05–2.35). A comparison among the age group revealed significant differences of using antipyretics (*p* < 0.05; in 104 (81.2%) participants in 20s, in 127 (69.4%) in 30s, in 115 (62.8%) in 40s, in 76 (63.9%) in 50s, and in 17 (47.2%) in 60s).

**TABLE 1:** Sex‐related and age‐related differences in systemic or local adverse reactions in the participants after the booster dose of the BNT162b2 messenger RNA vaccine (Pfizer‐BioNTech, Comirnaty)

	Systemic adverse reactions						Local adverse reactions				
	Fever (≧38 °C)	Headache	Malaise	Nausea	Myalgia	Arthralgia	Pain	Redness	Induration	Pruritus	Heat
Male, *n* (%) *N* = 141	26 (18.4)	68 (48.6)	103 (73.6)	7 (5.0)	71 (50.7)	36 (25.9)	127 (90.7)	13 (9.3)	14 (10.1)	12 (8.6)	40 (28.6)
Female, *n* (%) *N* = 508	114 (22.4)	317 (62.6)	405 (80.7)	82 (16.2)	327 (65.0)	242 (47.6)	472 (93.5)	95 (18.7)	51 (10.3)	85 (16.8)	169 (33.3)
*p* value	0.35	<0.05	0.08	<0.05	<0.05	<0.05	0.27	<0.05	1.00	<0.05	0.31
Odds ratio (95% CI)	1.29 (0.79–2.18)	1.77 (1.20–2.64)	1.50 (0.94–2.36)	3.66 (1.64–9.62)	1.8 (1.21–2.68)	2.60 (1.68–4.07)	1.46 (0.69–2.96)	2.25 (1.20–4.53)	1.02 (0.53–2.05)	2.13 (1.11–4.43)	1.25 (0.82–1.94)
20–29 years, *n* (%) *N* = 128	36 (30.8)	89 (69.5)	108 (84.4)	23 (18.0)	90 (70.9)	59 (46.1)	121 (94.5)	25 (19.5)	9 (7.2)	16 (12.5)	51 (39.8)
30–39 years, *n* (%) *N* = 183	45 (28.3)	112 (61.5)	150 (82.9)	24 (13.2)	121 (66.5)	79 (43.4)	171 (94.0)	30 (16.4)	14 (7.8)	29 (16.0)	63 (34.4)
40–49 years, *n* (%) *N* = 183	32 (19.2)	102 (55.7)	136 (74.7)	26 (14.3)	97 (53.9)	81 (44.3)	162 (89.0)	24 (13.1)	15 (8.4)	27 (14.8)	44 (24.0)
50–59 years, *n* (%) *N* = 119	22 (21.0)	73 (61.9)	93 (79.5)	13 (10.9)	71 (59.7)	50 (42.0)	113 (95.8)	26 (21.8)	22 (18.8)	19 (16.0)	43 (36.4)
≧60 years, *n* (%) *N* = 36	5 (13.9)	9 (25.7)	21 (61.8)	3 (8.3)	19 (54.3)	9 (25.7)	32 (91.4)	3 (8.8)	5 (14.7)	6 (17.6)	8 (22.9)
*p* value	<0.05	<0.05	<0.05	0.09	<0.05	0.13	0.61	0.59	0.97	0.47	0.07

*Note:* Data are presented as numbers (percentages).

Sex‐related differences in systemic or local adverse reactions were compared using Fisher's exact tests.

Age‐related differences in systemic or local adverse reactions were compared using Cochran–Armitage tests.

Abbreviation: CI, confidence interval.

This is a questionnaire‐based surveillance and a descriptive study. This study hypothesized that female and young participants might be more likely to have several adverse reactions and use antipyretics for the third dose, similar to the previous reports of the second and first doses.^3^ The higher frequency of several adverse reactions in the third dose was reported when compared to those in the second dose of vaccination, by Izumo et al.^3^ More information is needed on the possibility of adverse reactions because of the third dose of vaccination in the Japanese population.

## CONFLICT OF INTEREST

The authors have stated explicitly that there are no conflicts of interest in connection with this article.
